# Supplementary material for: Control of leucine-dependent mTORC1 pathway through chemical intervention of leucyl-tRNA synthetase and RagD interaction
Source: Nat Commun. 2017 Sep 29;8:732. doi: 10.1038/s41467-017-00785-0 (PMC5622079; doi:10.1038/s41467-017-00785-0)
Supplement: Supplementary file 1 — Supplementary Information [file 41467_2017_785_MOESM1_ESM.pdf]

Description of Supplementary Files

File Name: Supplementary Information
Description: Supplementary Figures and Supplementary Tables

File Name: Peer Review File

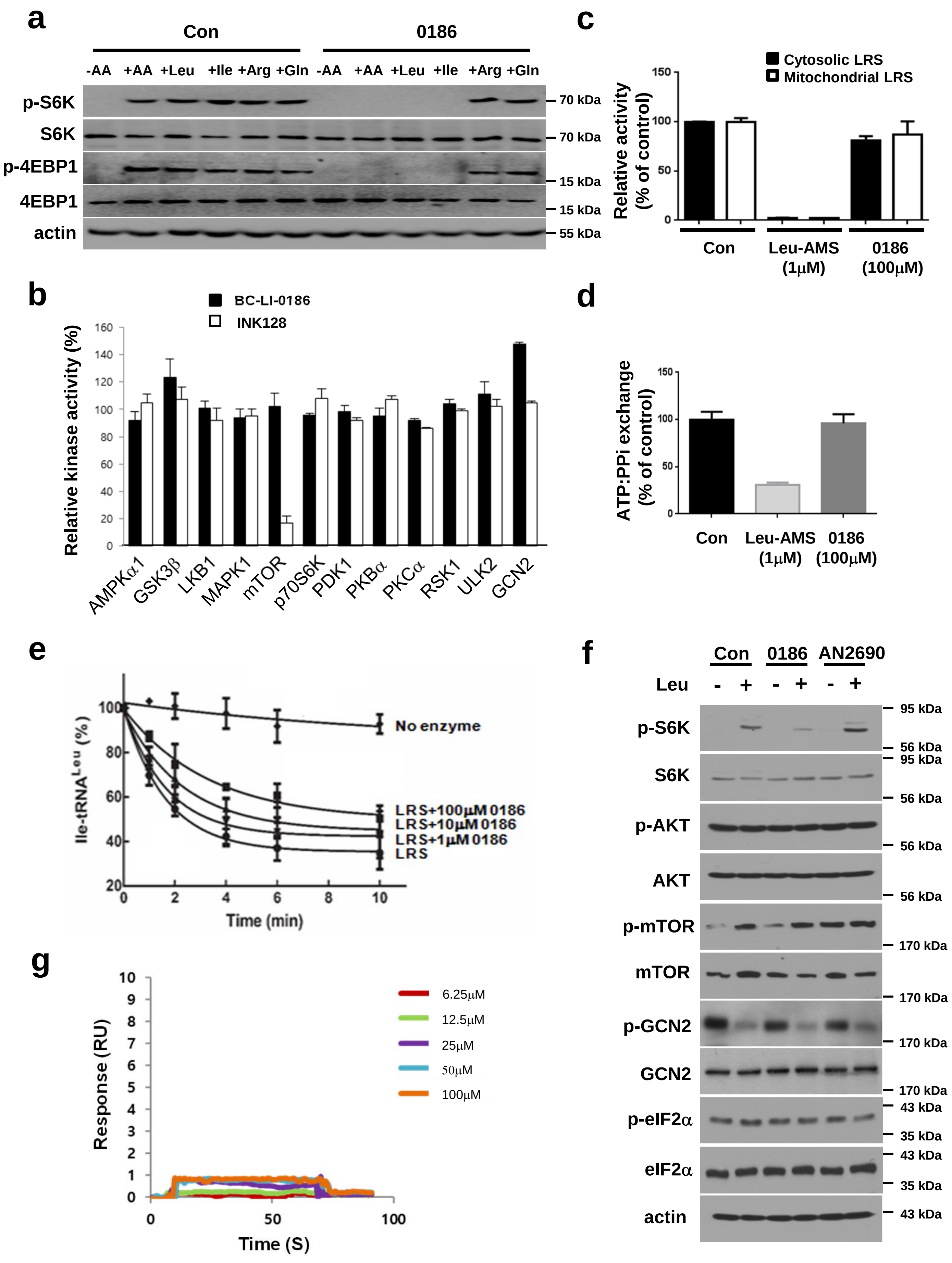
a
Con
0186
-AA +AA +Leu +Ile +Arg +Gln -AA +AA +Leu +Ile +Arg +Gln
p-S6K
S6K
p-4EBP1
4EBP1
actin
70 kDa
70 kDa
15 kDa
15 kDa
55 kDa
b
BC-LI-0186
INK128
Relative kinase activity (%)
AMPKα1 GSK3β LKB1 MAPK1 mTOR p70S6K PDK1 PKBα PKCα RSK1 ULK2 GCN2
c
Cytosolic LRS
Mitochondrial LRS
Relative activity (% of control)
Con
Leu-AMS (1μM)
0186 (100μM)
d
ATP:PPi exchange (% of control)
Con
Leu-AMS (1μM)
0186 (100μM)
e
Ile-tRNA^Leu (%)
No enzyme
LRS+100μM 0186
LRS+10μM 0186
LRS+1μM 0186
LRS
Time (min)
f
Con 0186 AN2690
Leu - + - + - +
p-S6K
S6K
p-AKT
AKT
p-mTOR
mTOR
p-GCN2
GCN2
p-eIF2α
eIF2α
actin
95 kDa
56 kDa
170 kDa
43 kDa
35 kDa
g
Response (RU)
Time (S)
6.25μM
12.5μM
25μM
50μM
100μM

Supplementary Figure 1. Effect of BC-LI-0186 on *in vitro* mTOR or LRS activity

a, Specific inhibition of leucine- and isoleucine-induced S6K phosphorylation by BC-LI-0186. Cells were starved amino acids for 90 min and re-stimulated for amino acids (10min), leucine (15min), isoleucine (15 min), glutamine (90min), or arginine (90 min) in the absence or presence of 10 μM BC-LI-0186. Cell lysates were analyzed by immunoblotting with the indicated antibodies. **b**, The *in vitro* kinase panel assay was performed with BC-LI-0186 (10 μM) or INK128 (100 nM), known as an mTOR kinase inhibitor. mTOR kinase was inhibited by INK128 used as a positive control. **c**, Leucylation assay was performed in the presence of Leu-AMS (1 μM) or BC-LI-0186 (100 μM) with 50 nM purified human cytosolic or 2.5 μM mitochondrial LRS (LRS2). Data are means±S.D. (n=3). **d**, ATP:PPi exchange activity assay was carried out in the presence of Leu-AMS (1 μM) or BC-LI-0186 (100 μM) with 50 nM purified human cytosolic LRS. Data are means±S.D. (n=3). **e**, Deacylation activity of mischarged $tRNA^{Leu}$ by human cytosolic LRS in the presence or absence of BC-LI-0186. No Enzyme (◆; diamond), No BC-LI-0186 (●; circle), 1 μM BC-LI-0186 (▼; inverted triangle), 10 μM BC-LI-0186 (▲; triangle), 100 μM BC-LI-0186 (■; square). The error bars represent mean±S.D. (*n* = 3). **f**, Cells were treated with 10 μM BC-LI-0186 or 50 μM AN2690. Cell lysates were analyzed by immunoblotting with the indicated antibodies. **g**, The binding of BC-LI-0186 to SESN2 was determined by surface plasmon resonance (SPR). The compound was injected to immobilized Sestrin2 at the indicated concentrations and the binding was measured using Biacore T200. The response data were processed using data from a reference and buffer injections.

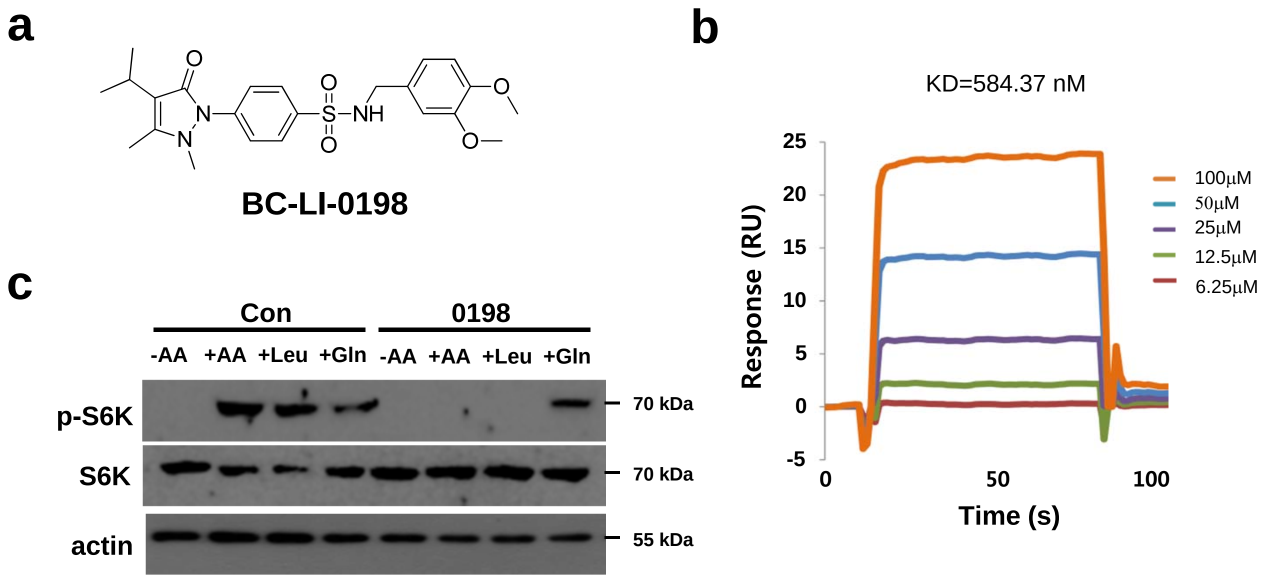

a
BC-LI-0198
b
KD=584.37 nM
Response (RU)
Time (s)
100μM
50μM
25μM
12.5μM
6.25μM
c
Con
0198
-AA
+AA
+Leu
+Gln
p-S6K
S6K
actin
70 kDa
70 kDa
55 kDa

Supplementary Figure 2. Direct binding of BC-LI-0198 with LRS

a, Chemical structure of BC-LI-0198. **b,** The binding of BC-LI-0198 to LRS WT was determined by surface plasmon resonance (SPR) as described in Methods. **c**, Specific inhibition of leucine-induced S6K phosphorylation by BC-LI-0186. Cells were starved amino acids, leucine, or glutamine for 90 min and re-stimulated for amino acids (10min), leucine (15min), or glutamine (90 min) in the absence or presence of 10 μM BC-LI-0186. Cell lysates were analyzed by immunoblotting with the indicated antibodies.

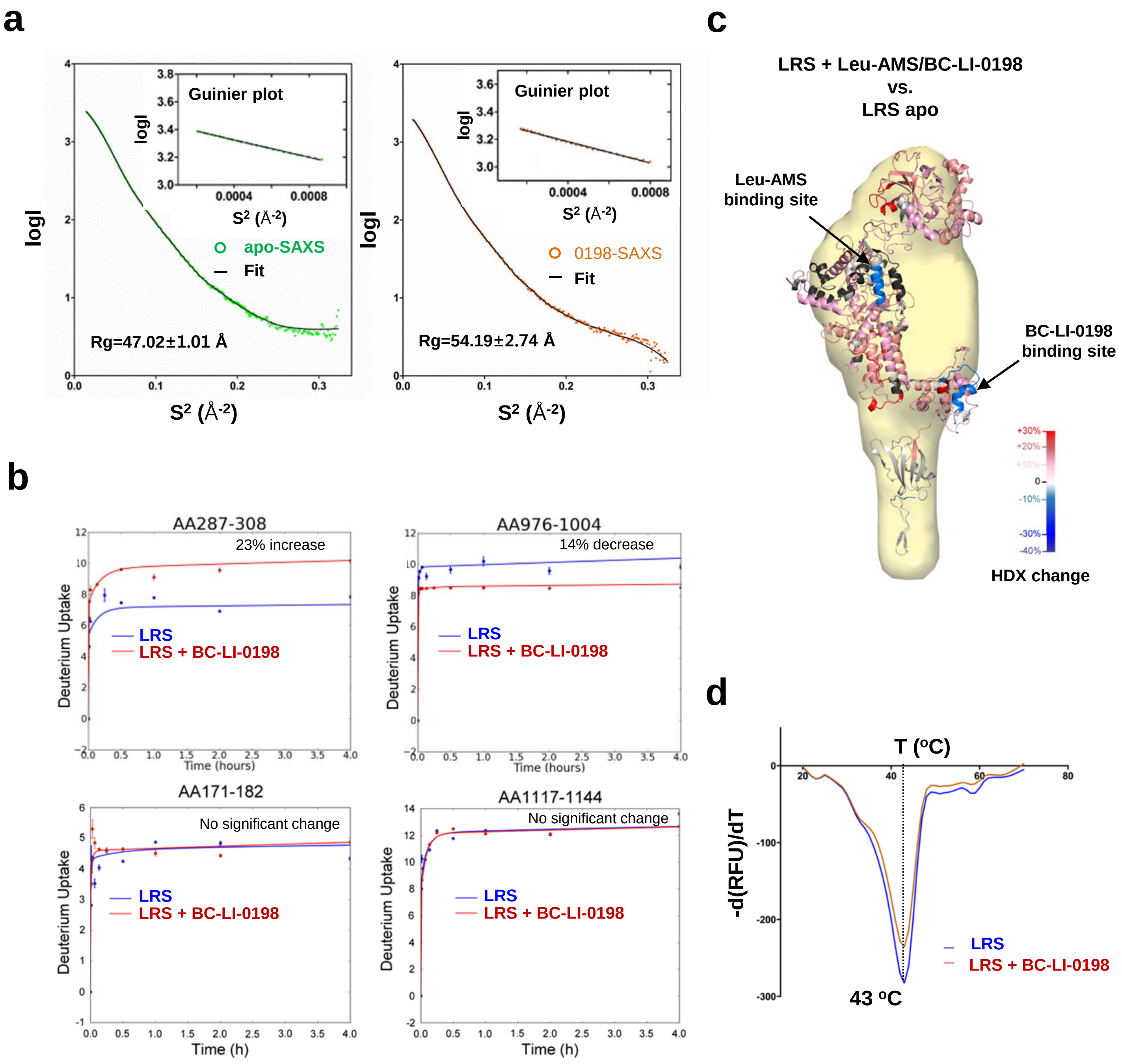
a
Guinier plot
logI
S² (Å⁻²)
apo-SAXS
Fit
Rg=47.02±1.01 Å
0198-SAXS
Rg=54.19±2.74 Å
b
AA287-308
23% increase
AA976-1004
14% decrease
AA171-182
No significant change
AA1117-1144
Deuterium Uptake
Time (hours)
Time (h)
LRS
LRS + BC-LI-0198
c
LRS + Leu-AMS/BC-LI-0198
vs.
LRS apo
Leu-AMS
binding site
BC-LI-0198
binding site
HDX change
d
T (ºC)
-d(RFU)/dT
43 ºC

Supplementary Figure 3. Mapping of BC-LI-0198 docking site

a, SAXS data analysis of LRS apo (left) and LRS with BC-LI-0198 (right). The theoretical scattering profiles calculated from the *ab initio* models of the two experimental groups with the lowest χ values are shown in black lines. The insets represent the Guinier plots at the low angle region ($S_{max}R_g < 1.3$), revealing no aggregation in the samples. **b**, HDX-MS analysis of LRS (blue) and LRS with BC-LI-0198 (red). HDX time curves showed more solvent exposure of residues aa 287-308 (upper left) and less solvent exposure of residues aa 976-1004 (critical region of Rag complex binding) (upper right). The peptides show no difference of deuterium uptake upon BC-LI-0198 binding (lower left and lower right). **c**, HDX-MS and SAXS analysis of LRS with Leu-AMS/BC-LI-0198. Structural model of LRS with Leu-AMS/BC-LI-0198, which was modified manually from the predicted prototype model, was docked in SAXS envelop. Colors indicate changes of deuterium uptake compared with LRS apo as shown in the gradient color bar. **d**, Thermal shift curves of LRS apo (blue), and LRS with BC-LI-0198 (orange).

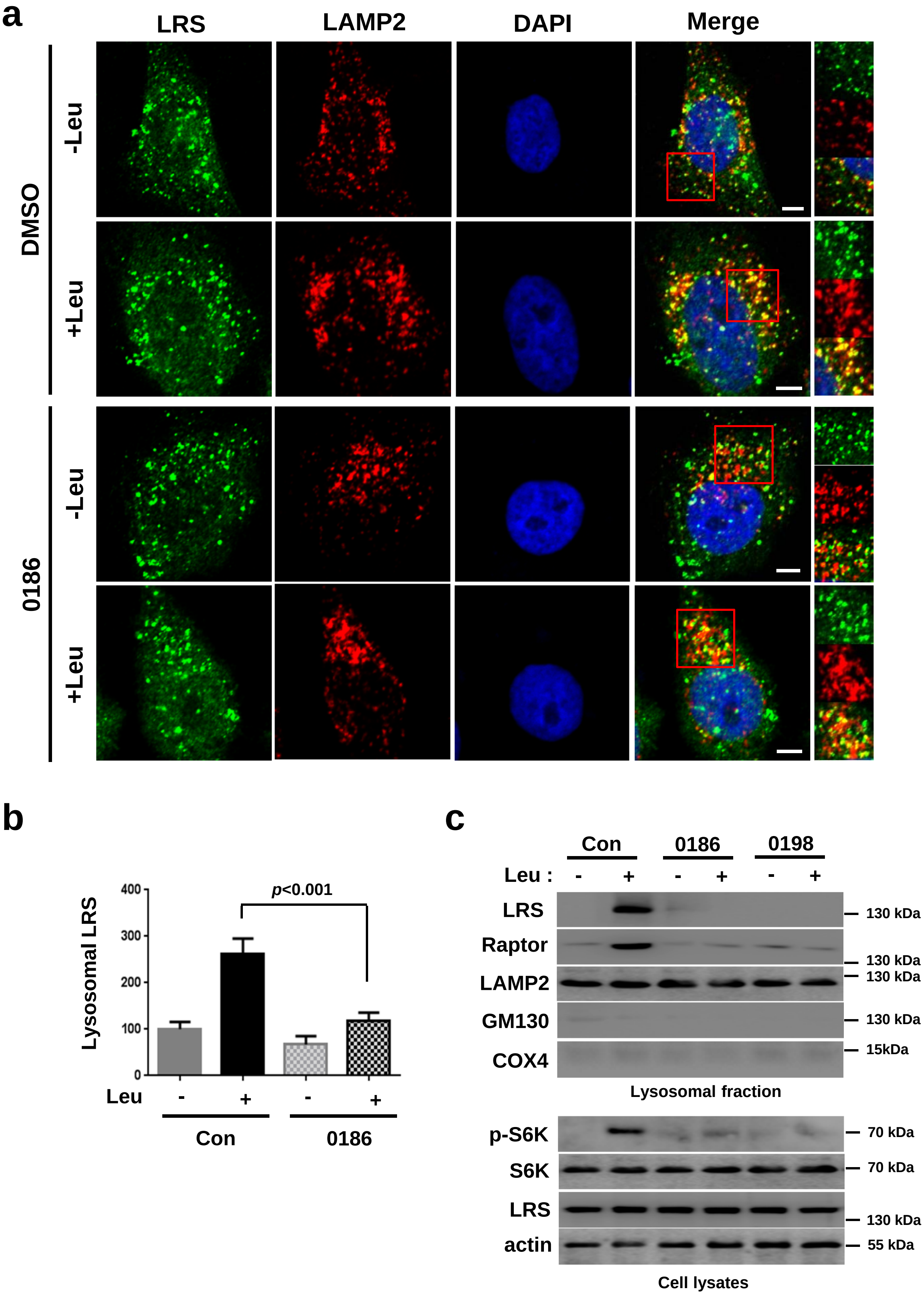
a
LRS
LAMP2
DAPI
Merge
DMSO
-Leu
+Leu
0186
-Leu
+Leu
b
Lysosomal LRS
400
300
200
100
0
p<0.001
Leu
-
+
-
+
Con
0186
c
Con
0186
0198
Leu : - + - + - +
LRS
Raptor
LAMP2
GM130
COX4
130 kDa
130 kDa
130 kDa
130 kDa
15kDa
Lysosomal fraction
p-S6K
S6K
LRS
actin
70 kDa
70 kDa
130 kDa
55 kDa
Cell lysates

Supplementary Figure 4. Effect of BC-LI-0186 on lysosomal localization of LRS and Raptor

a, Co-localization of LRS with lysosomal marker LAMP2 in SW620 cells. Cells were starved leucine for 90 min and re-stimulated with leucine for 15 min in the presence or absence of 10 μM BC-LI-0186. Cells were reacted with anti-LRS and anti-LAMP2 antibodies and visualized with alexa 488-conjugated and alexa 594-conjugated secondary antibodies, respectively. Scale bar = 5μm. **b**, Quantification of the co-localization between LRS and LAMP2 was performed by using the colocalization function of ImageJ. The index of co-localization corresponds to the mean of the overlap coefficient (R)*100 obtained for more than 20 cells for each co-labeling. The ratio between green and red signals is comprised between 0.8 and 1.2. (p<0.001). **c**, Effect of BC-LI-0186 and BC-LI-0198 on lysosomal localization of LRS and Raptor. SW620 cells were starved leucine for 90 min and re-stimulated with leucine for 15 min in the presence or absence of 10 μM BC-LI-0186 or BC-LI-0198. Lysosomal proteins were immunoblotted with anti-LRS, anti-Raptor, and anti-LAMP2 antibodies. GM130 and COX4 were used as Golgi and mitochondrial markers, respectively.

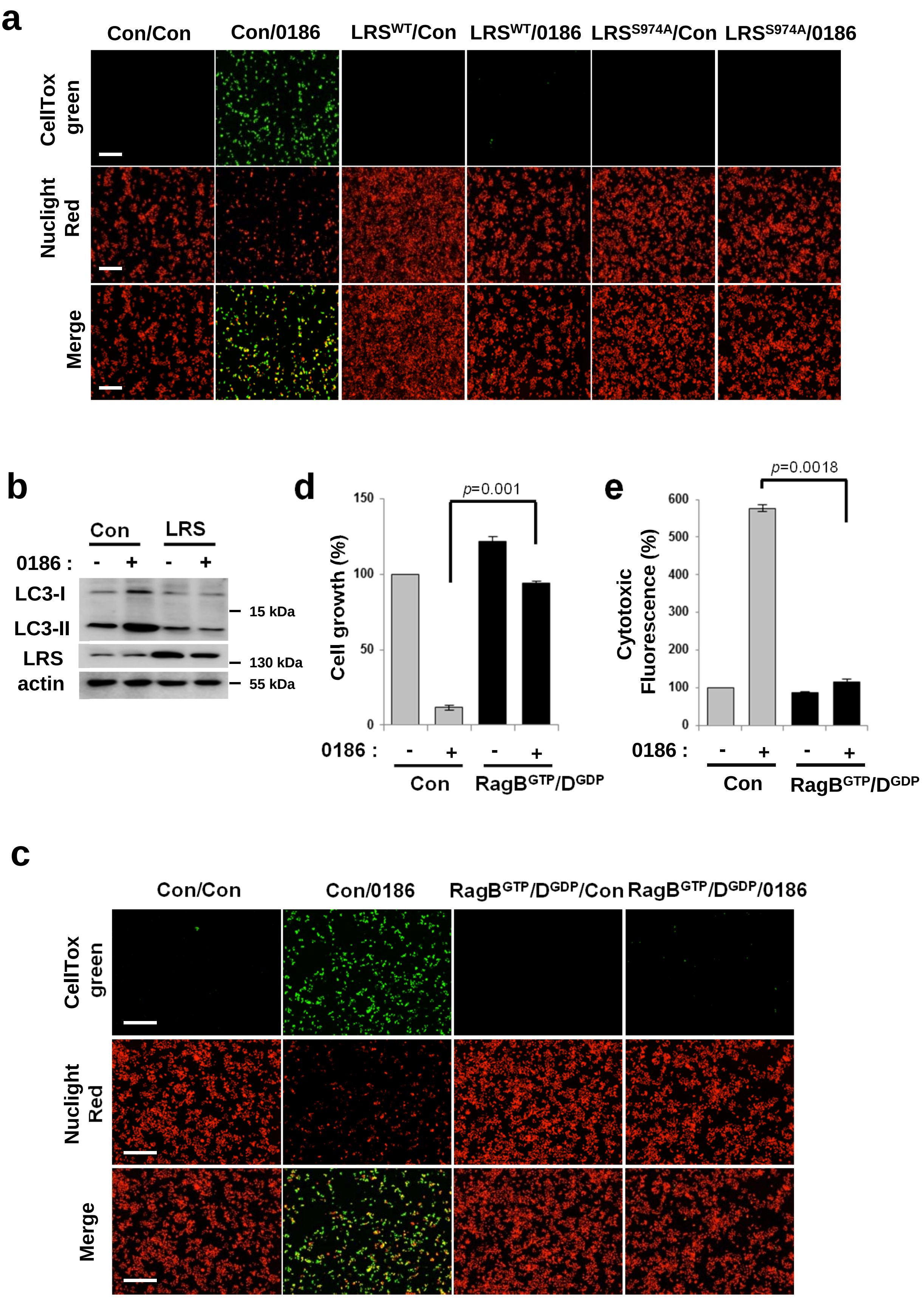
a
Con/Con
Con/0186
LRSWT/Con
LRSWT/0186
LRSS974A/Con
LRSS974A/0186
CellTox green
Nuclight Red
Merge
b
Con
LRS
0186 :
LC3-I
LC3-II
LRS
actin
15 kDa
130 kDa
55 kDa
d
Cell growth (%)
p=0.001
0186 :
Con
RagBGTP/DGDP
e
Cytotoxic Fluorescence (%)
p=0.0018
0186 :
Con
RagBGTP/DGDP
c
Con/Con
Con/0186
RagBGTP/DGDP/Con
RagBGTP/DGDP/0186
CellTox green
Nuclight Red
Merge

Supplementary Figure 5. Compensation of BC-LI-0186 activity with LRS and RagD

a, Effect of LRS WT or S974A overexpression on BC-LI-0186-induced growth inhibition and cell death. Inducible LRS-overexpressed SW620 cells were untreated (Con) or treated with DOX for 72 hr (LRS) and then with 10 μM BC-LI-0186 in the presence of CellTox green to detect cell death. After 24 hr incubation, cell images were acquired. Representative images were shown. Scale bar = 300μm. **b**, Inducible WT LRS-overexpressed SW620 cells were untreated (Con) or treated with DOX for 72 hr (LRS) and then with 10 μM BC-LI-0186 for 24 hr. Cellular levels of LC3-I, -II, LRS, and actin were monitored by immunoblotting with their specific antibodies. **c**, Effect of $RagB^{GTP}/D^{GDP}$ overexpression on BC-LI-0186-induced growth inhibition and cell death. SW620 cells stably expressing nuclear RFP were transfected with EV or Myc-$RagB^{GTP}$/HA-$RagD^{GDP}$. After 24 hr, the cells were treated with BC-LI-0186 (10 μM) in the presence of CellTox green to monitor cell death. Representative images were shown. Scale bar = 300μm. **d-e**, Effect of $RagB^{GTP}/D^{GDP}$ overexpression on BC-LI-0186-induced growth inhibition (**d**) and cell death (**e**). The data in (**c**) were quantified and displayed as bar graphs. The error bars represent mean±S.D. (*n* = 3).

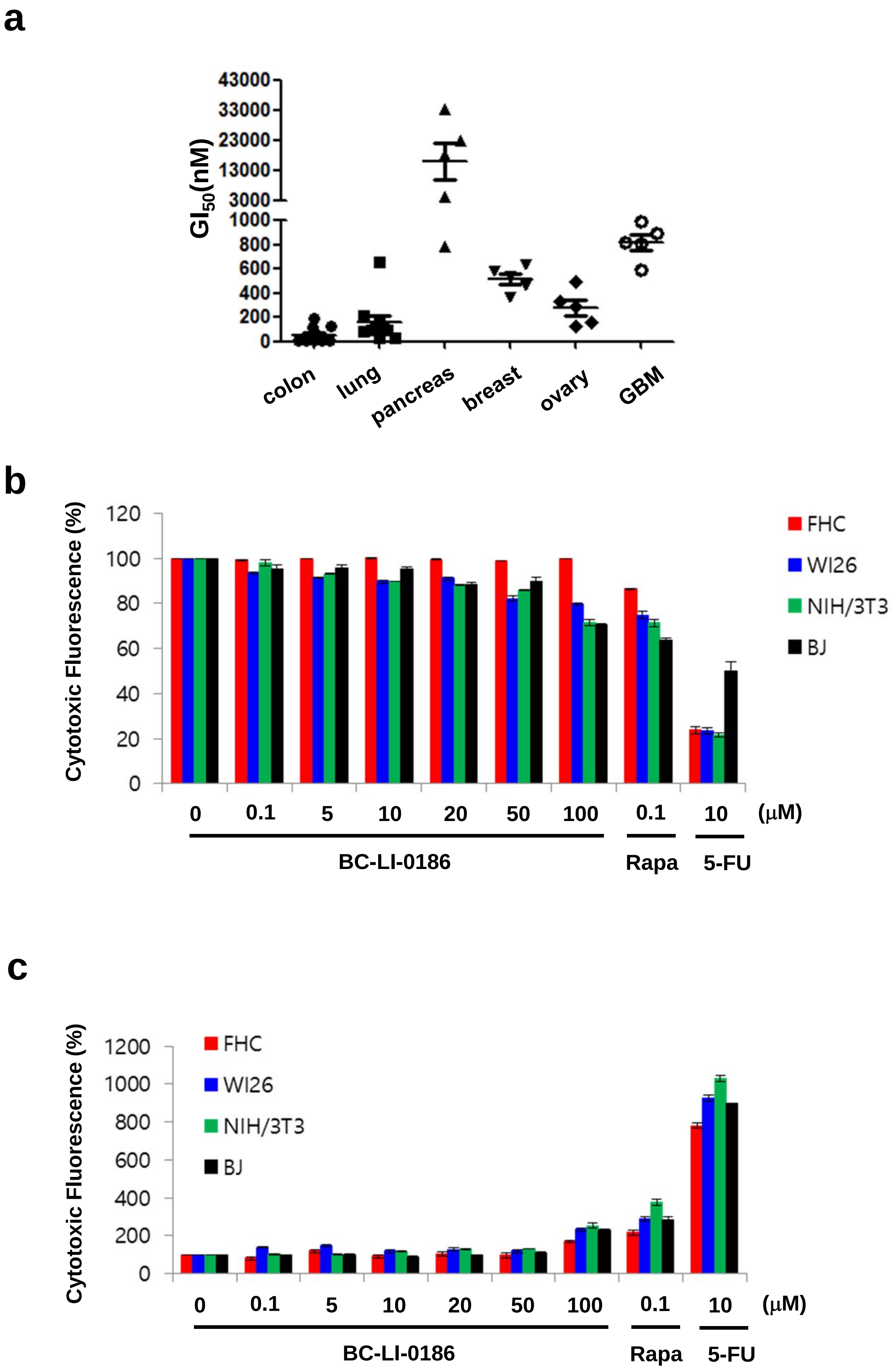
a
GI50(nM)
43000
33000
23000
13000
3000
1000
800
600
400
200
0
colon
lung
pancreas
breast
ovary
GBM
b
Cytotoxic Fluorescence (%)
120
100
80
60
40
20
0
FHC
WI26
NIH/3T3
BJ
0
0.1
5
10
20
50
100
0.1
10
(μM)
BC-LI-0186
Rapa
5-FU
c
Cytotoxic Fluorescence (%)
1200
1000
800
600
400
200
0
FHC
WI26
NIH/3T3
BJ
0
0.1
5
10
20
50
100
0.1
10
(μM)
BC-LI-0186
Rapa
5-FU

Supplementary Figure 6. Activity of BC-LI-0186 in other cancer cells and normal cells

a, GI_{50} values of BC-LI-0186 against 13 colon cancer cells, 11 lung cancer, 5 pancreatic cancer, 5 breast cancer, 5 ovarian cancer, and 5 glioblastoma cancer are shown. The error bars represent mean±S.D. **b**, Normal cells were treated with rapamycin (100 nM), 5-FU (10 μM) or BC-LI-0186 at the indicated concentration. After 24hr, cell growth was measured. **c**, Normal cells were treated with rapamycin (Rapa, 100 nM), 5-FU (10 μM), or BC-LI-0186 at the indicated concentration in the presence of CellTox green. After 24 hr, cell death was measured via analysis of green fluorescence. The error bars represent mean±S.D. (*n* = 3).

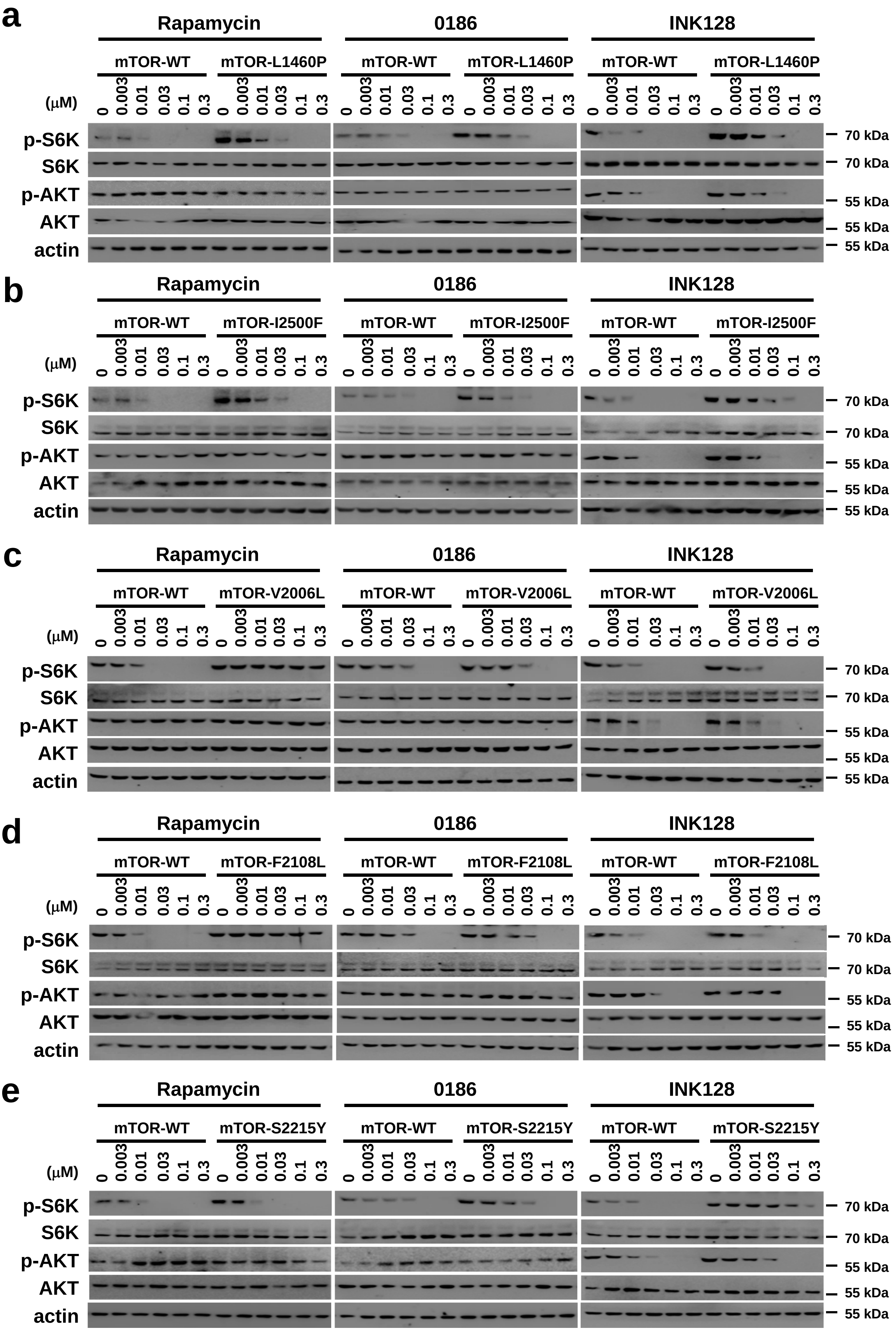

a
Rapamycin
0186
INK128
mTOR-WT
mTOR-L1460P
(μM)
0
0.003
0.01
0.03
0.1
0.3
p-S6K
S6K
p-AKT
AKT
actin
70 kDa
70 kDa
55 kDa
55 kDa
55 kDa
b
Rapamycin
0186
INK128
mTOR-WT
mTOR-I2500F
(μM)
0
0.003
0.01
0.03
0.1
0.3
p-S6K
S6K
p-AKT
AKT
actin
70 kDa
70 kDa
55 kDa
55 kDa
55 kDa
c
Rapamycin
0186
INK128
mTOR-WT
mTOR-V2006L
(μM)
0
0.003
0.01
0.03
0.1
0.3
p-S6K
S6K
p-AKT
AKT
actin
70 kDa
70 kDa
55 kDa
55 kDa
55 kDa
d
Rapamycin
0186
INK128
mTOR-WT
mTOR-F2108L
(μM)
0
0.003
0.01
0.03
0.1
0.3
p-S6K
S6K
p-AKT
AKT
actin
70 kDa
70 kDa
55 kDa
55 kDa
55 kDa
e
Rapamycin
0186
INK128
mTOR-WT
mTOR-S2215Y
(μM)
0
0.003
0.01
0.03
0.1
0.3
p-S6K
S6K
p-AKT
AKT
actin
70 kDa
70 kDa
55 kDa
55 kDa
55 kDa

Supplementary Figure 7. BC-LI-0186 suppresses the activity of cancer-associated *MTOR* mutants

SW620 cells were transfected with FLAG-tagged mTOR WT, L1460P (**a**), I2500F (**b**), V2006L (**c**), F2108L (**d**), or S2215Y (**e**). Cells were treated with the indicated concentrations of rapamycin, BC-LI-0186, or INK128 for 6 hr. Cell lysates were analyzed with the indicated antibodies.

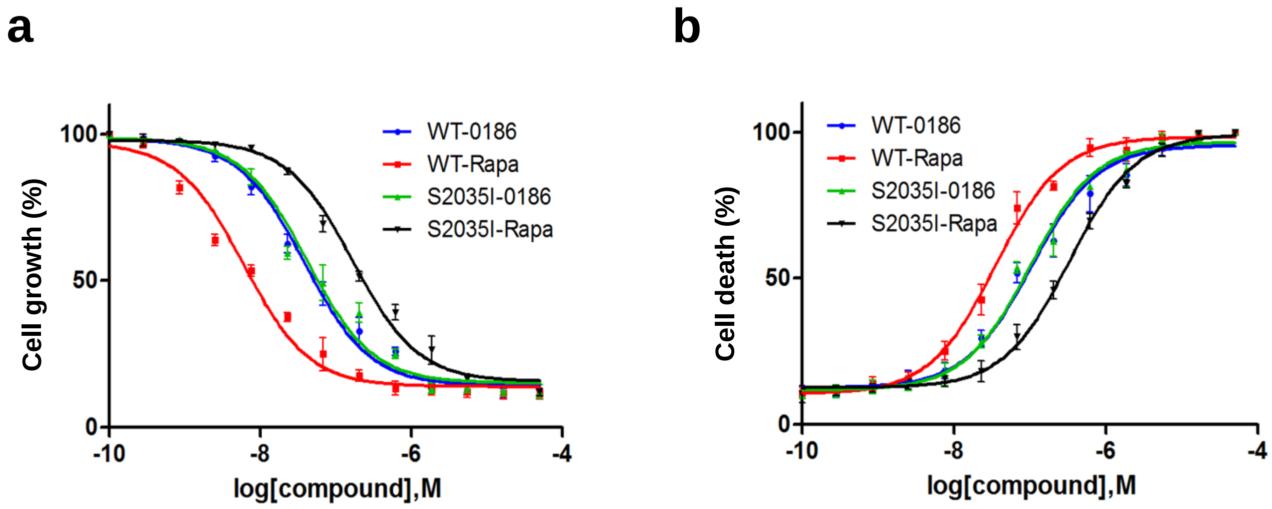
a
b
Cell growth (%)
Cell death (%)
log[compound],M
WT-0186
WT-Rapa
S2035I-0186
S2035I-Rapa
100
50
0
-10
-8
-6
-4

Supplementary Figure 8. BC-LI-0186 suppresses the growth of HCT116 MM cells

a, HCT116 cells expressing $mTOR^{WT}$ (MW) or isogenic $mTOR^{S2035I}$- (MM) were treated with BC-LI-0186 or rapamycin. After 24 hr, cell growth was measured as described in Methods. The error bars represent mean±S.D. (n = 3). **b**, HCT116 MW and MM cells were treated with BC-LI-0186 and rapamycin. After 24 hr, cell death was measured using CellTox green. The error bars represent mean±S.D. (n = 3).

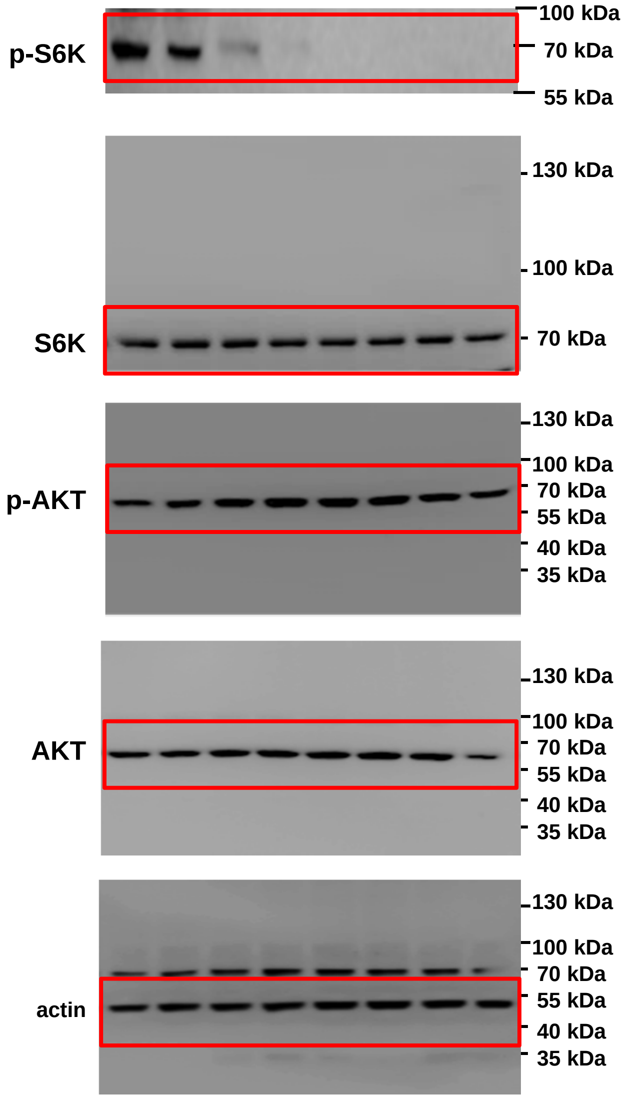

Fig. 1f

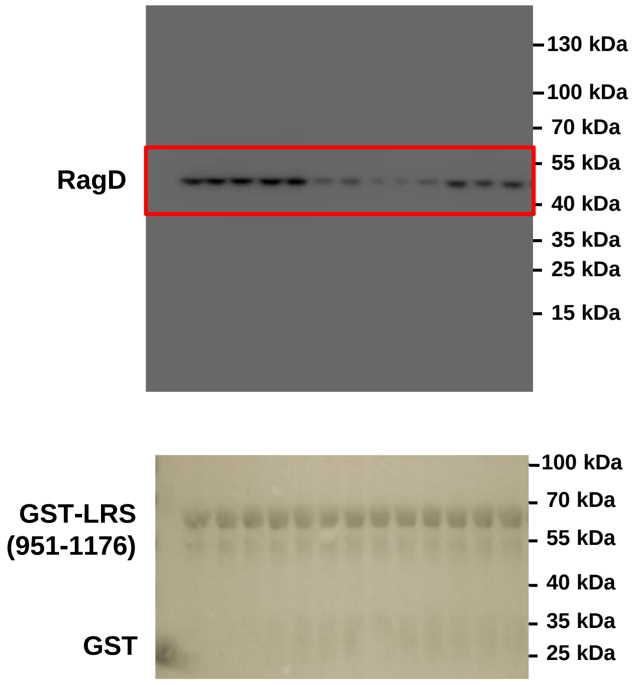

Fig. 3a

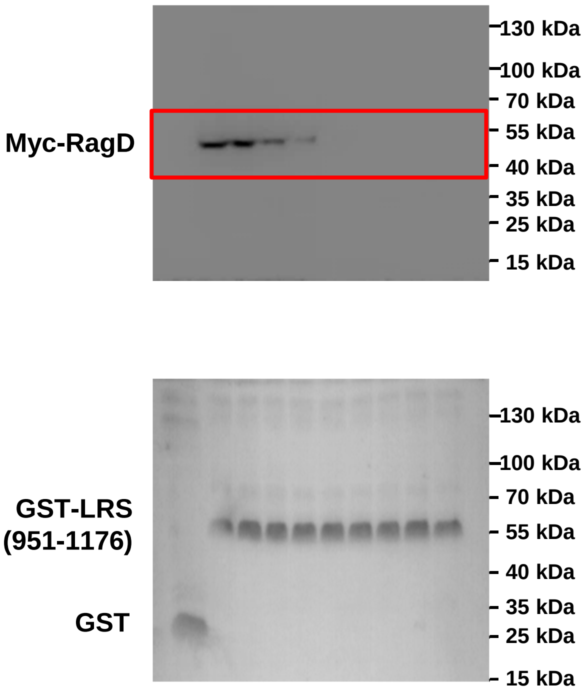

Fig. 3b

Supplementary Figure 9. Original western blot images

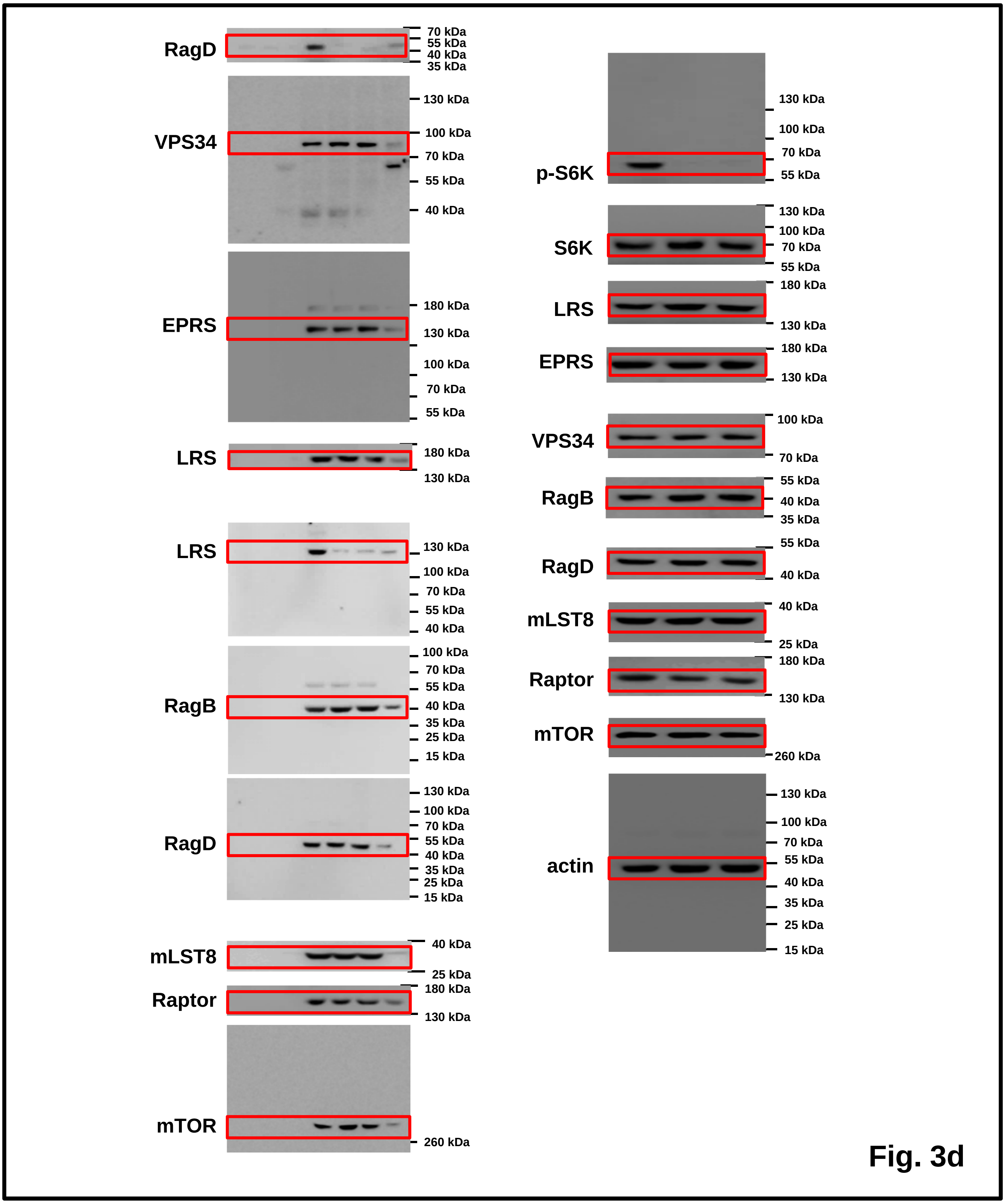

Supplementary Figure 9. Continued.

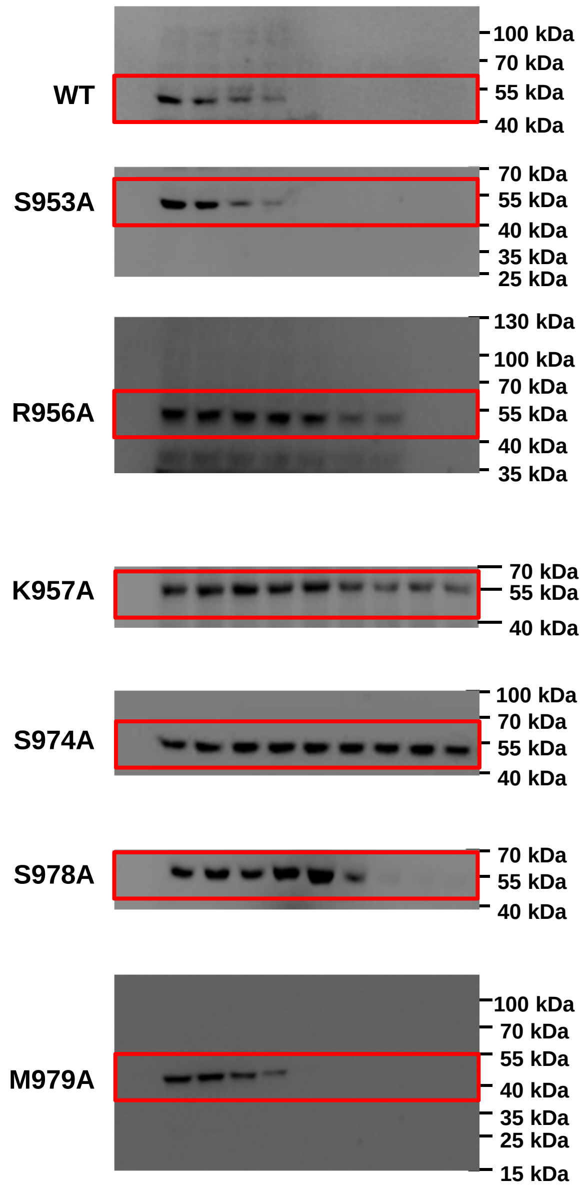

Fig. 3e

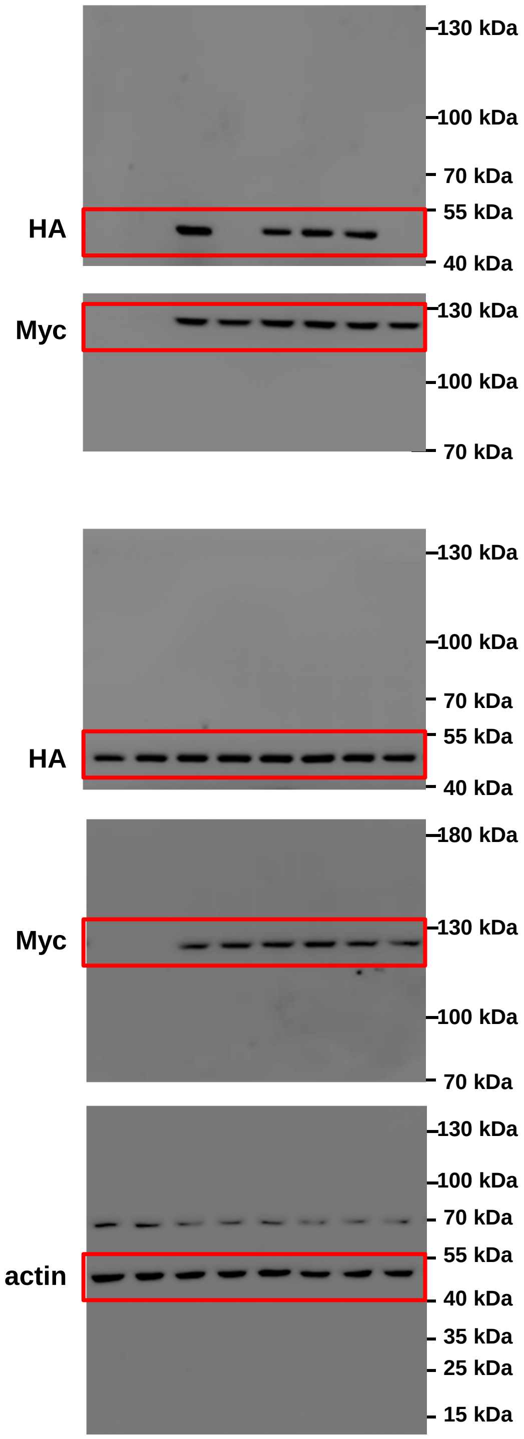

Fig. 3f

Supplementary Figure 9. Continued.

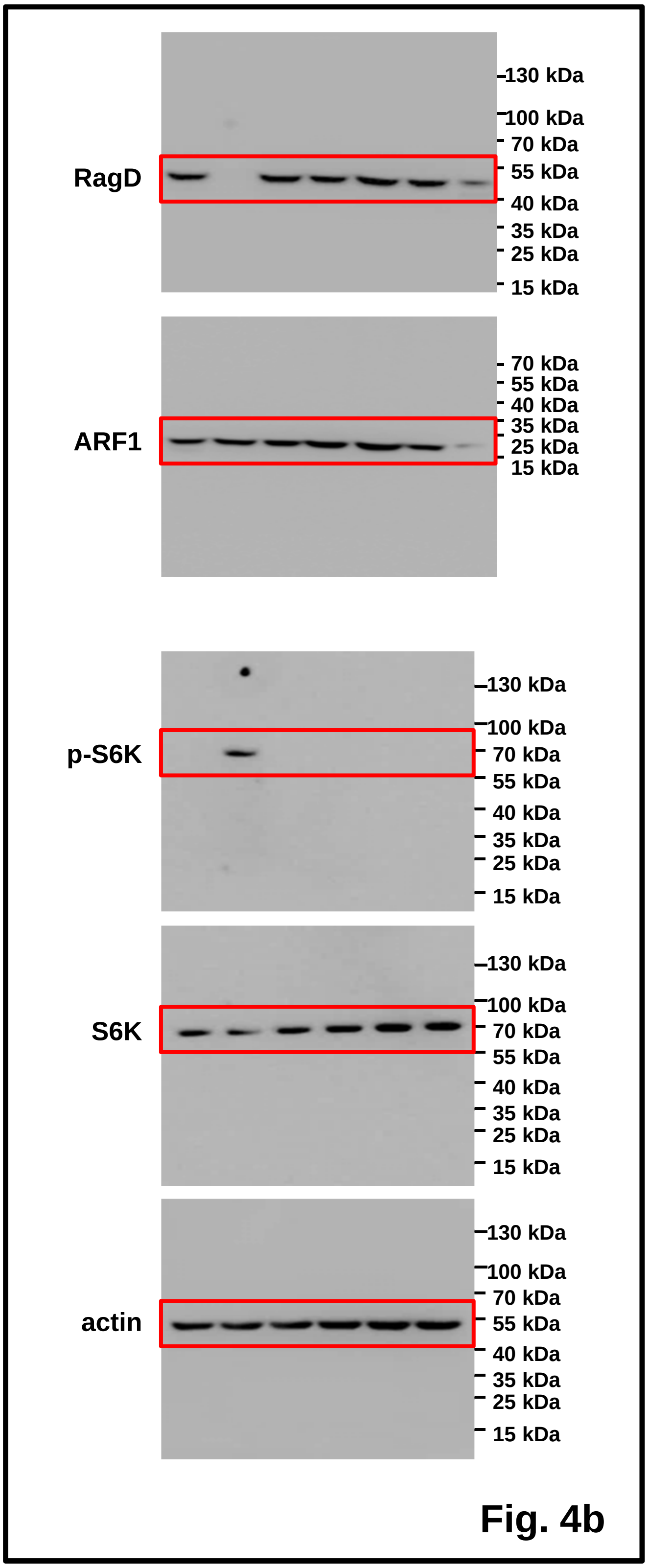

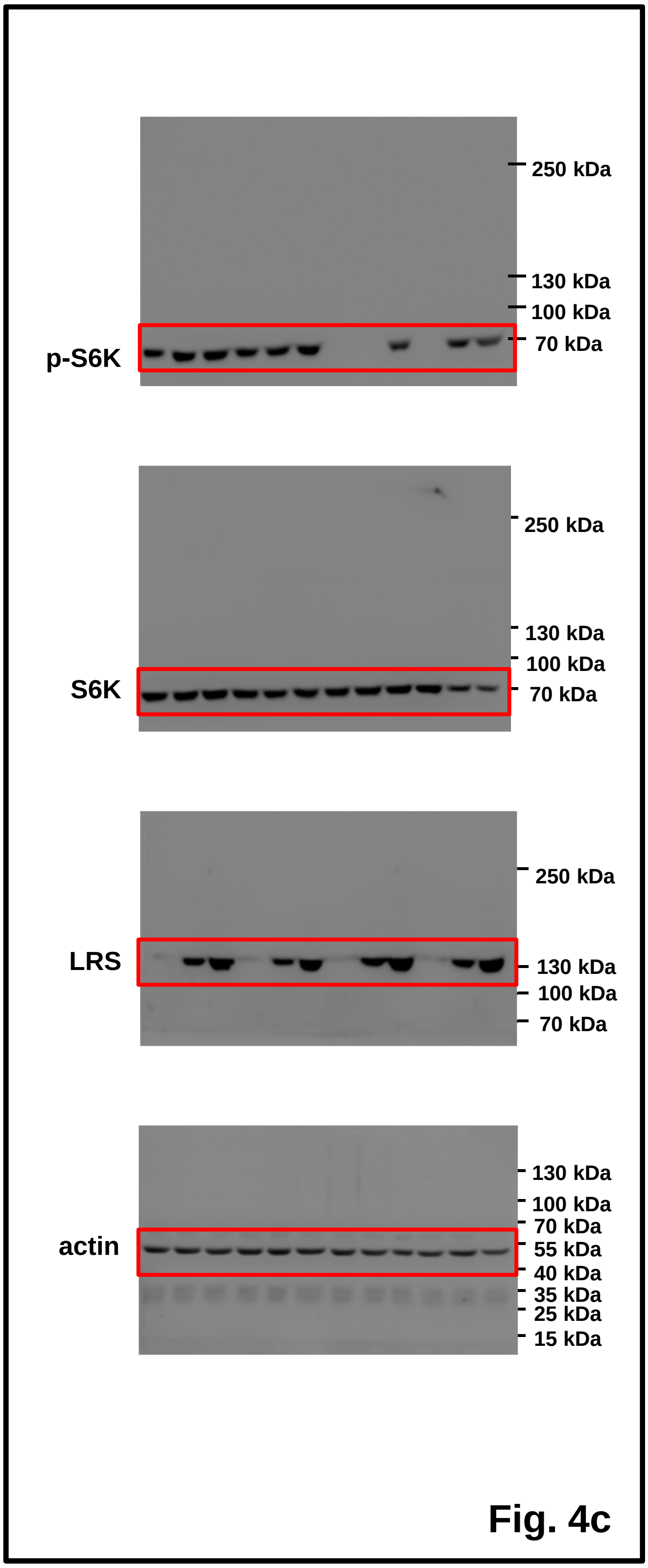

Supplementary Figure 9. Continued.

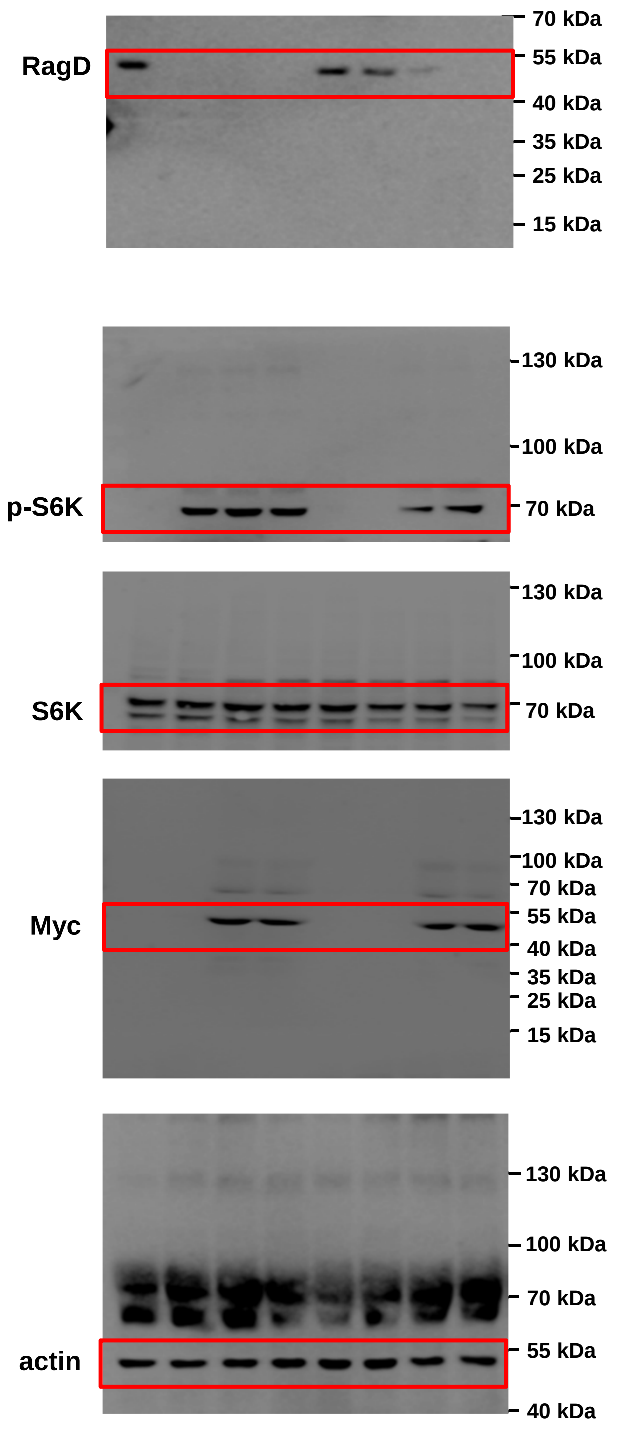

Fig. 4f

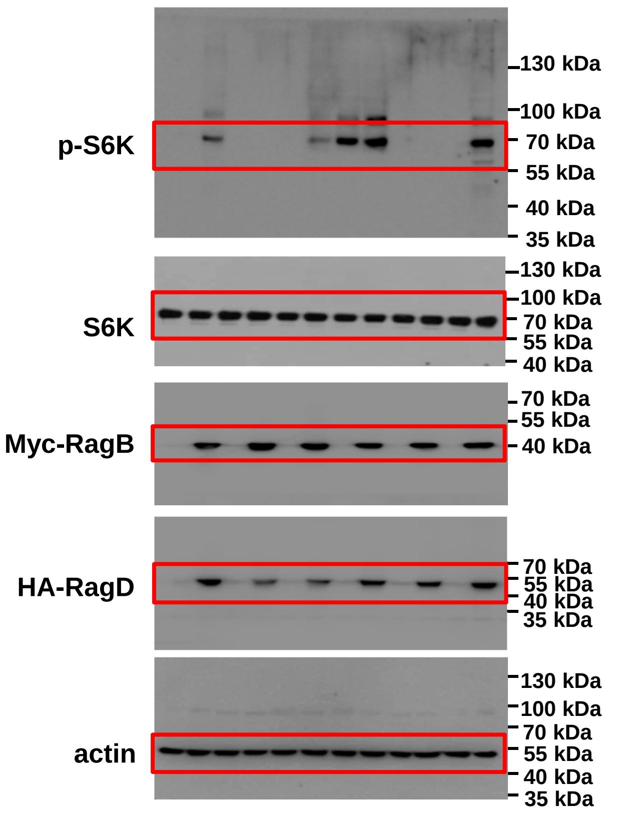

Fig. 4g

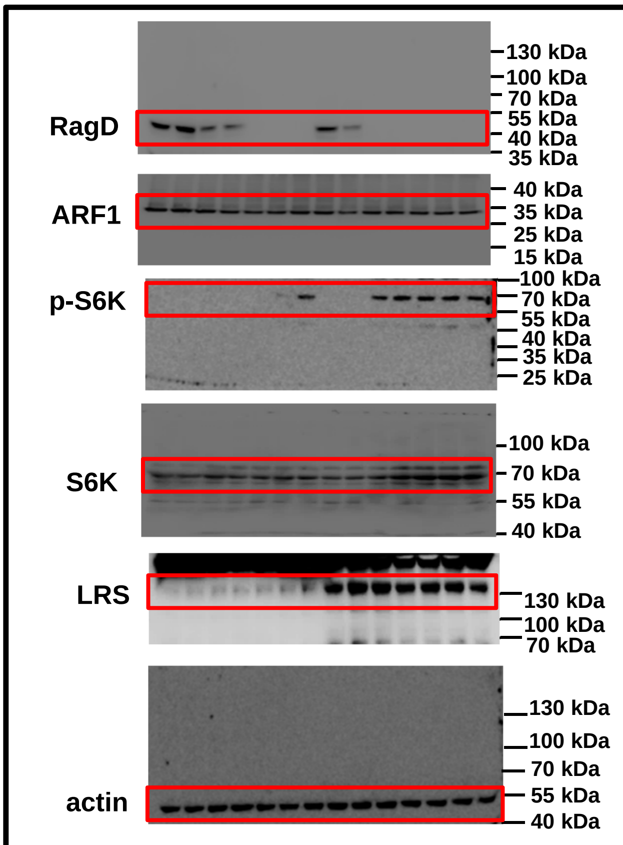

Fig. 4h

Supplementary Figure 9. Continued.

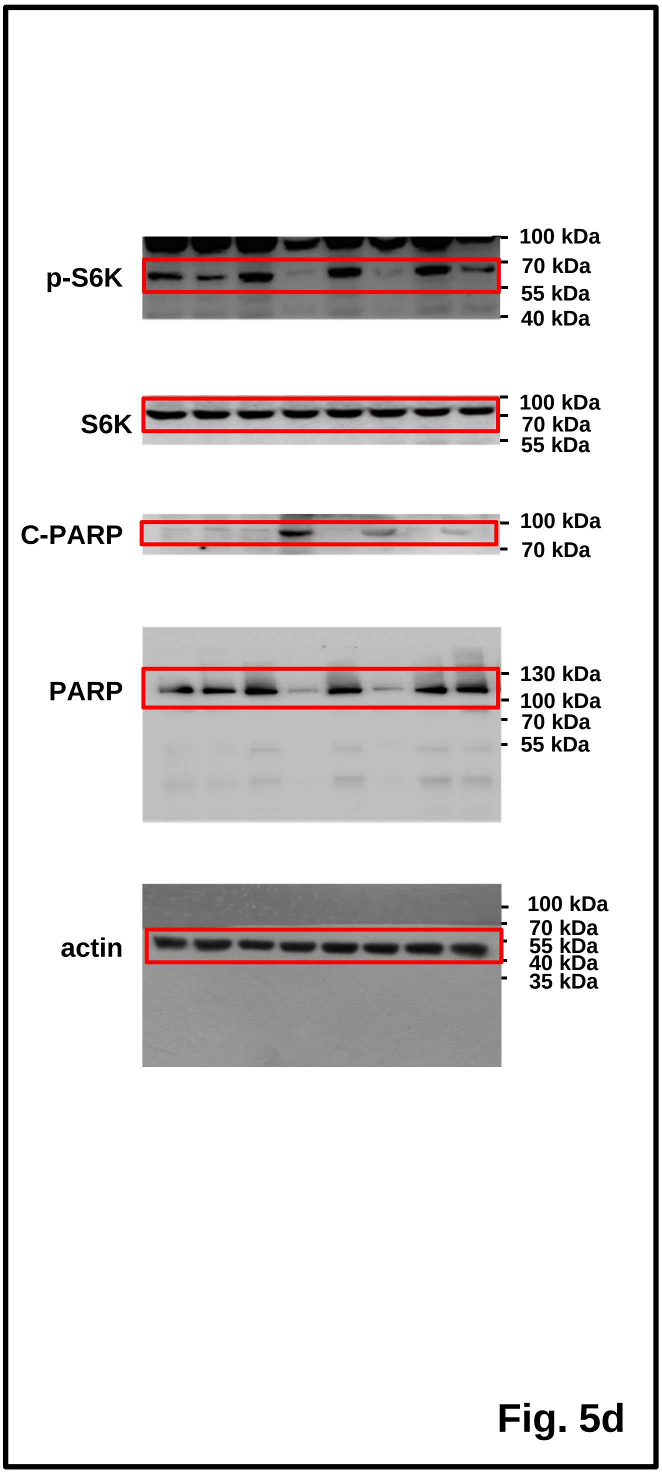

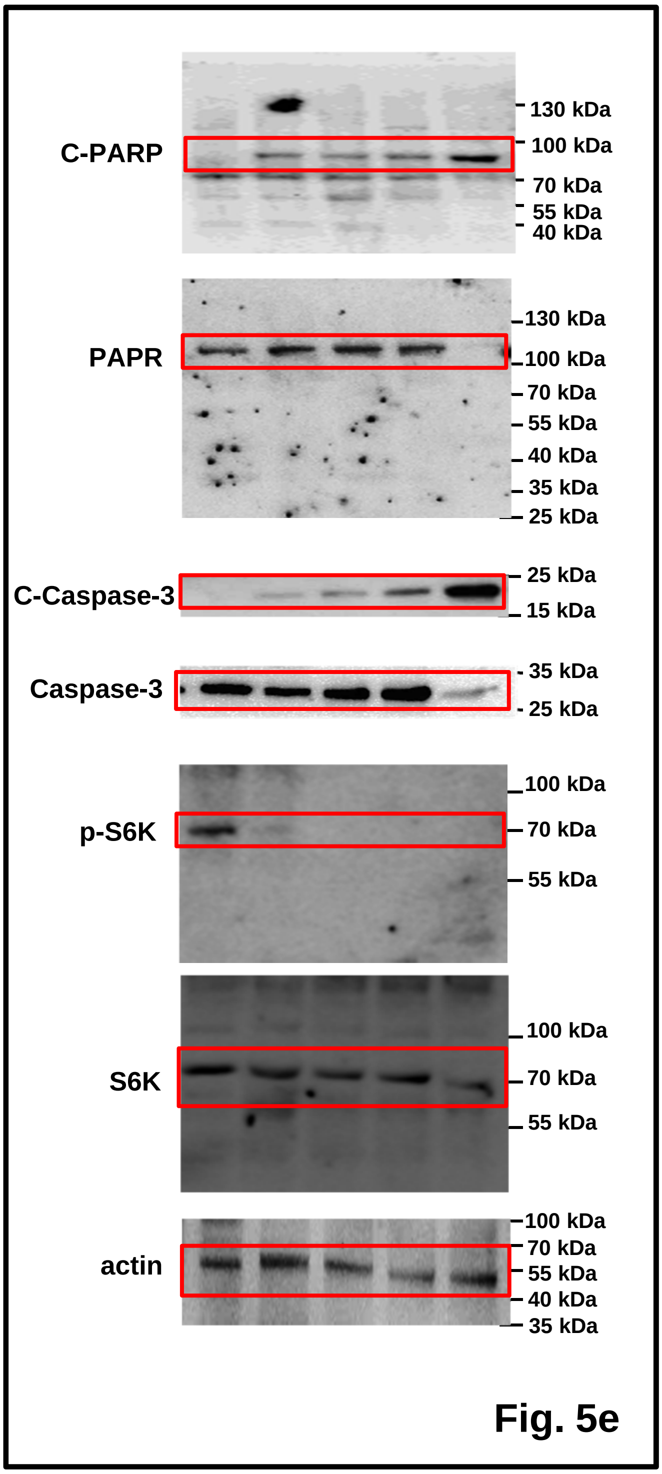

Supplementary Figure 9. Continued.

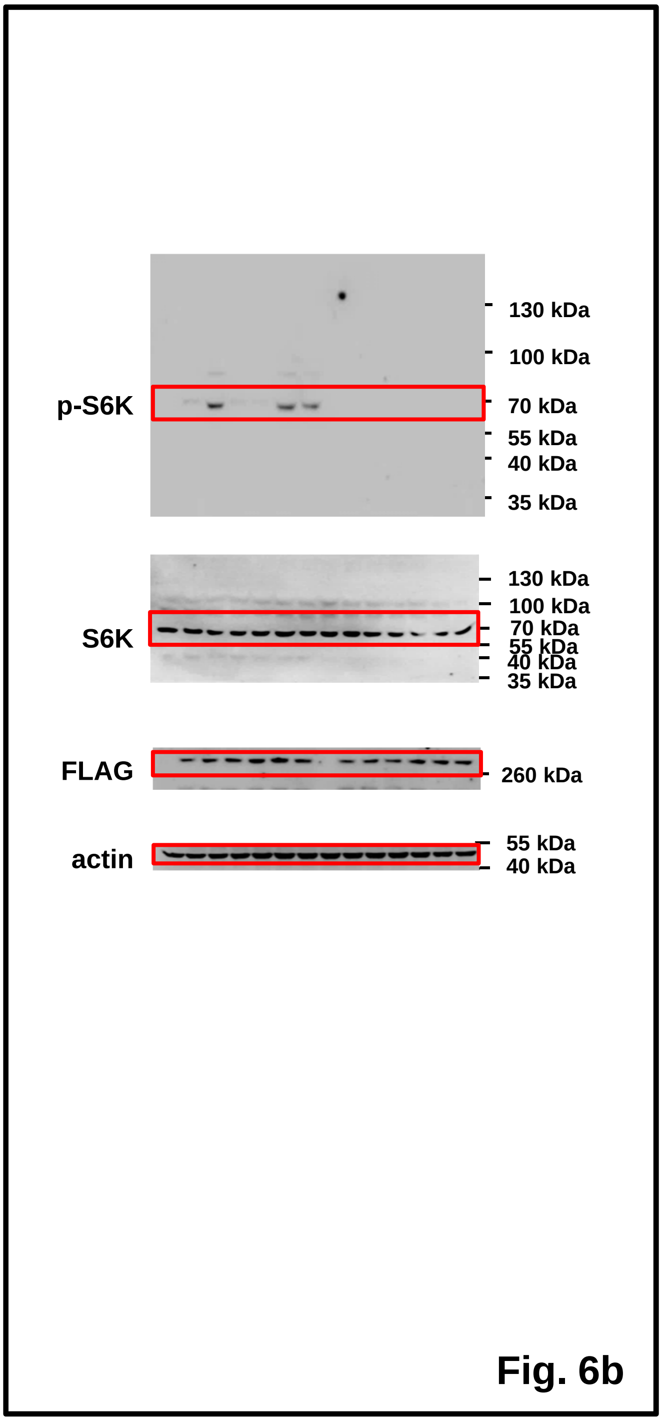

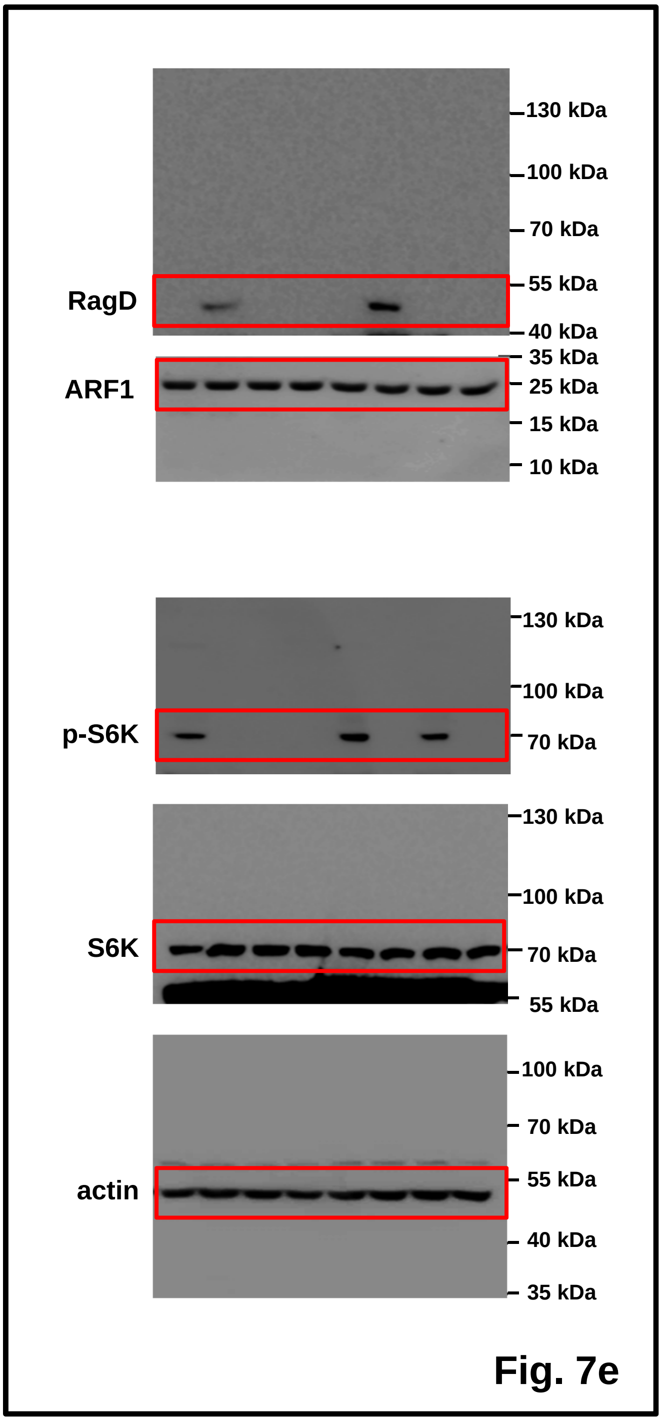

Supplementary Figure 9. Continued.

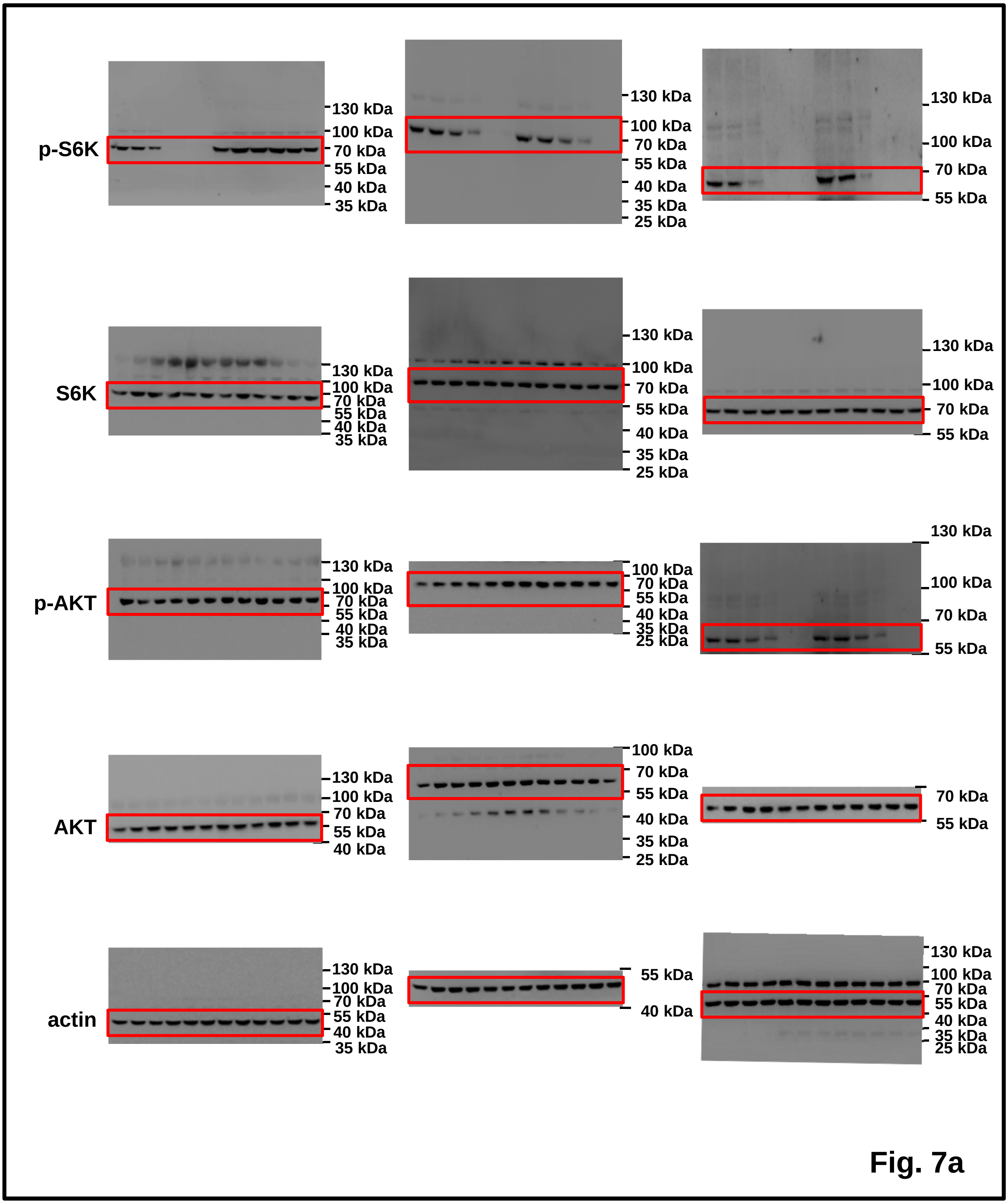

Supplementary Figure 9. Continued.

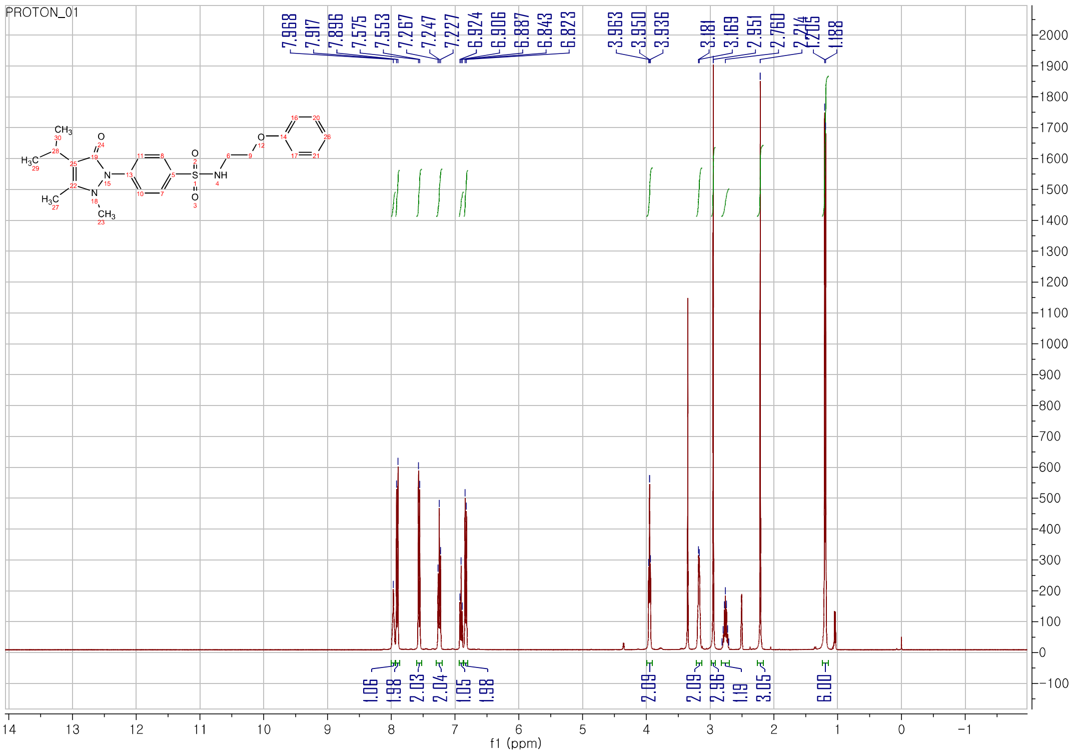

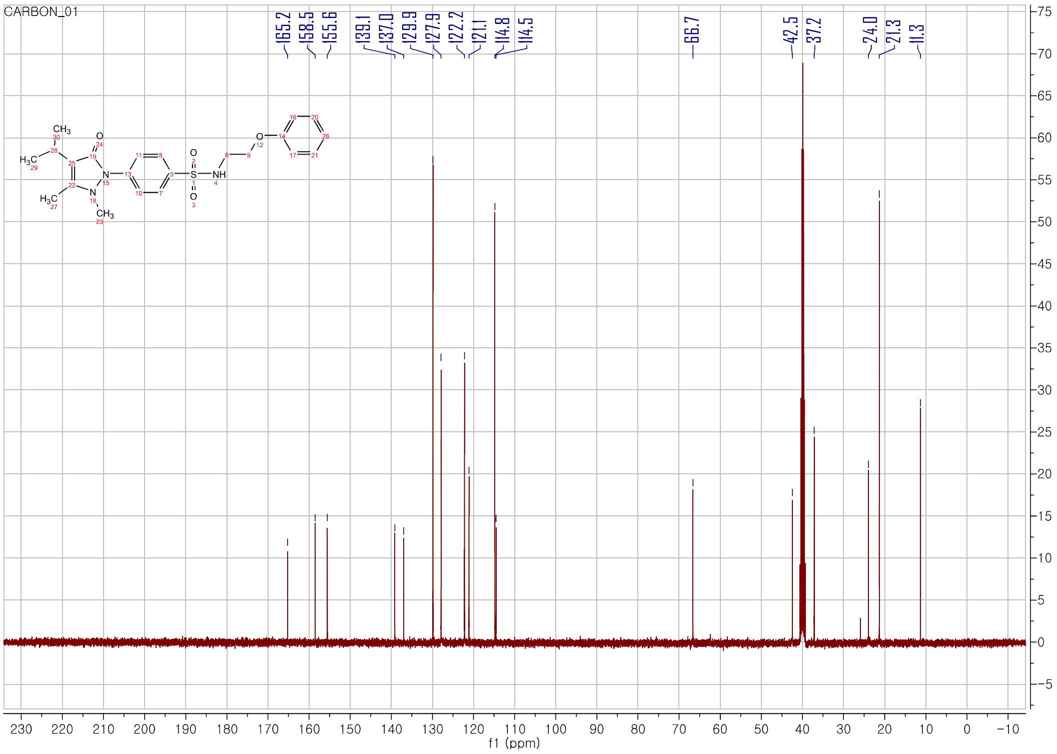

Supplementary Figure 10. ^{1}H (up) and ^{13}C (down) NMR spectrum of BC-LI-0186.

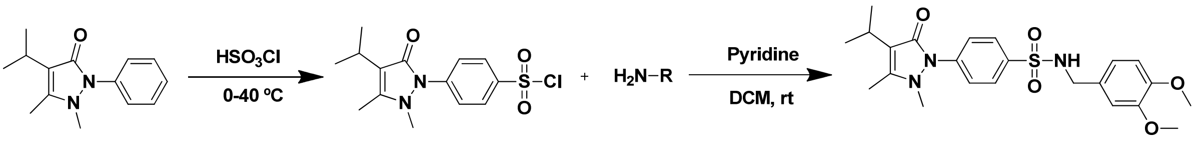

1 2

Supplementary Figure 11. Scheme of Chemical synthesis of BC-LI-0198

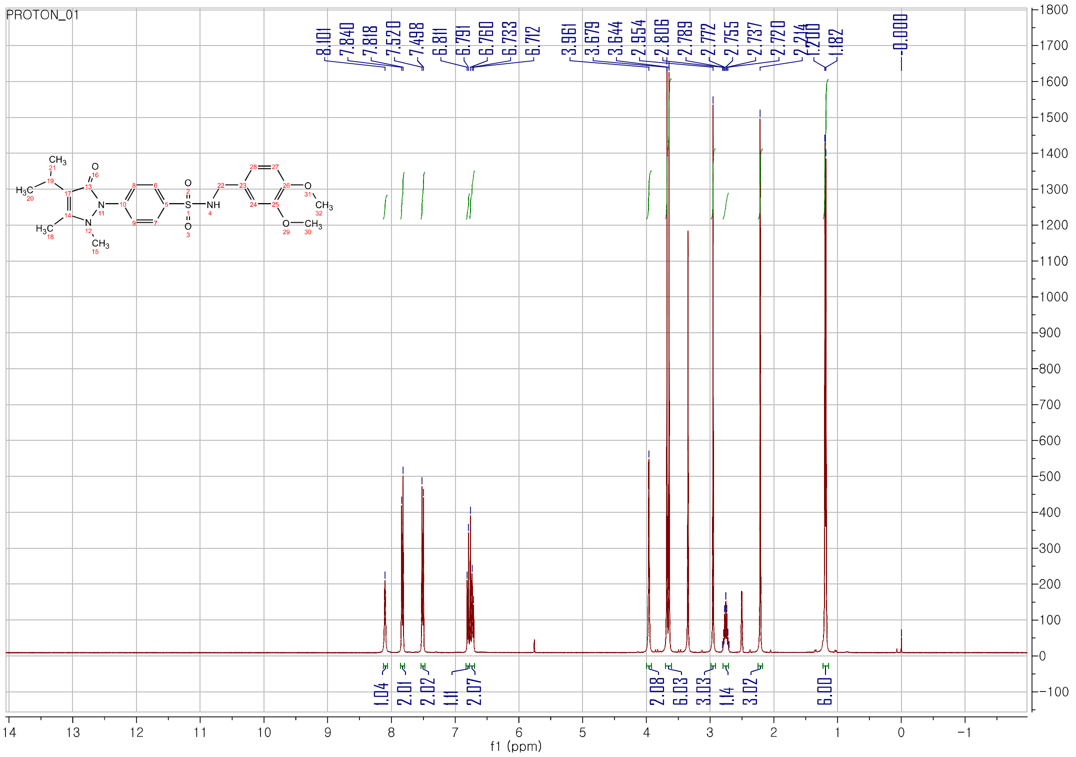

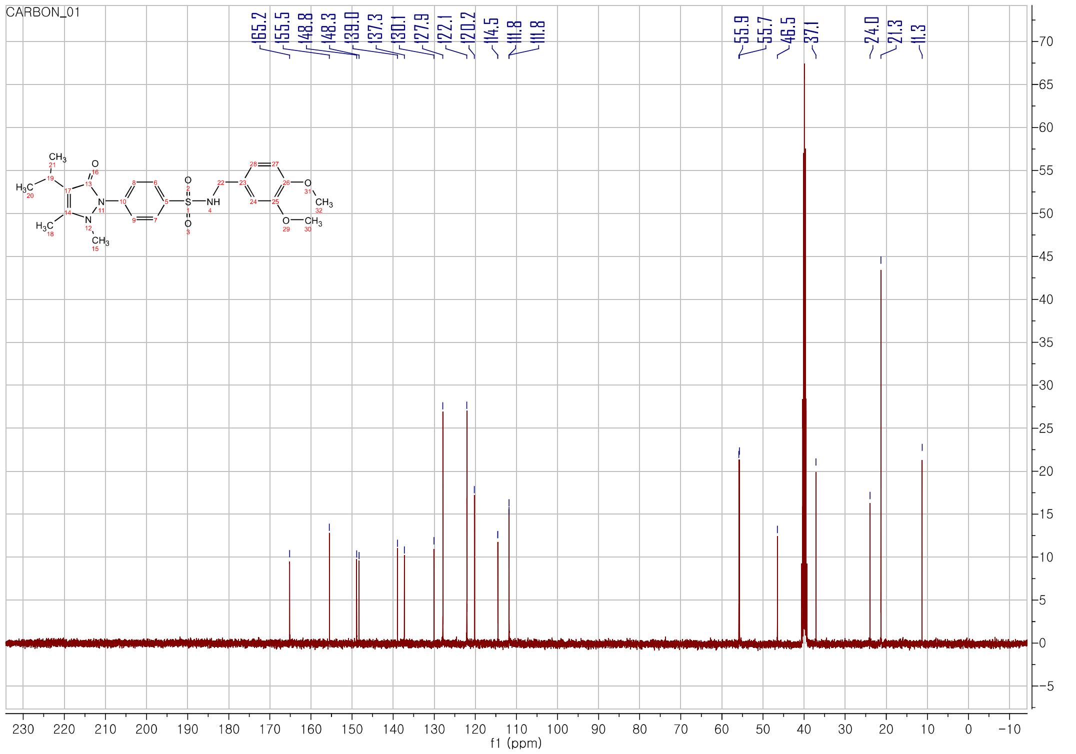

Supplementary Figure 12. ^{1}H (up) and ^{13}C (down) NMR spectrum of BC-LI-0198.

Supplementary Table 1. Summary of selected compounds

Compound	Leucine-dependent mTORC1 activity inhibition (% , at 20μM)	mTORC1 activity (IC_{50}, nM)	Cell Growth (GI_{50}, nM)	Cell Death (EC_{50}, nM)	Normal cell Viability (%, at 100 μM)	Solubility (μg/ml)		Physicochemical profiles	
						In PBS	In 3DW	AlogP	tPSA
BC-LI-0176	74.75±0.62	275±24	108±1.89	403±0.78	84.626±2.707	ND	2.88	2.507	85.44
BC-LI-0185	73.44±0.64	397±92	791±4.89	1297±0.26	97.788±3.036	ND	ND	1.177	78.54
BC-LI-0186	85.60±1.97	109.4±8.1	11±0.97	62.±3.48	97.8±2.53	3.41	3.62	2.916	87.32
BC-LI-0187	89.54±1.80	136±11	761±2.12	1127±1.32	79.654±9.982	ND	ND	3.888	87.32
BC-LI-0188	71.57±0.80	1623±311	729±0.93	1028±0.96	94.018±2.653	ND	ND	2.156	78.1
BC-LI-0198	68.53±4.6	1657±42	400.2±3.85	688.6±10.60	94.582±1.47	374.93	423.81	2.834	96.56
BC-LI-0225	78.06±1.35	574.5±193	37.8±2.26	358.7±9.74	90.566±1.963	3.95	3.93	2.646	96.56
BC-LI-0226	78.59±3.49	561±86.5	364.7±3.74	525.3±21.39	92.001±2.369	1.38	2.02	3.022	81.34
BC-LI-0227	77.08±3.26	521.7±22.4	247.3±4.93	423.1±6.59	94.423±1.557	2.19	1.26	2.716	104.4
BC-LI-0228	76.97±0.37	123.2±2	136.2±4.27	358.8±2.77	102.937±2.496	1.51	1.37	3.064	104.4
BC-LI-0252	94.18±3.81	73.9±5	20.5±0.87	204.4±6.22	80.303±2.02	4.33	4.52	3.833	78.1
BC-LI-0276	77.52±12.94	1030.7±23.1	340.3±1.52	912.6±5.49	89.402±1.820	145.06	174.98	2.834	96.56
BC-LI-0279	79.89±11.32	286±8	86.4±2.18	321.9±3.39	91.898±1.771	>500	104.37	2.583	90.99
BC-LI-0293	85.54±4.67	411.6±34	42.85±2.14	103.99±5.67	88.070±1.64	4.12	4.07	4.189	78.1
BC-LI-0298	88.83±13.38	465.8±32	25.70±5.14	141.91±7.99	95.628±2.08	2.50	0.58	3.73	78.1
BC-LI-0305	82.67±0.58	428.4±86	60.12±9.02	120.23±11.06	95.417±2.97	<2.05	<2.05	3.776	78.1
BC-LI-0321	85.3±2.66	146±5	27.93±2.79	108.64±15.24	93.528±3.22	3.98	4.23	3.675	78.1
BC-LI-0323	82.81±2.05	630.1±25	30.20±4.53	103.75±4.98	94.153±1.66	3.09	3.06	4.131	78.1
BC-LI-0326	79.65±1.06	214.8±14	19.91±3.98	158.85±4.16	89.626±2.35	4.39	4.78	3.803	78.1
BC-LI-0327	80.38±1.92	188±29	70.79±2.18	137.2±3.39	98.215±1.68	4.13	4.07	2.653	96.56
BC-LI-0328	89.84±12.77	288±67	15.24±0.91	44.74±1.75	95.038±1.76	8.449	8.496	2.13	87.33

ND: not determined

Supplementary Table 2. Comparison of growth inhibitory effects of BC-LI-0186, Rapamycin, and INK128 in mTOR WT or mutant-overexpressed SW620 cells

mTOR type	Growth inhibition (GI_{50}, nM)		
	0186	Rapamycin	INK128
WT	39.01±1.23	6.81±1.90	4.21±0.91
L1460P	41.89±1.29	7.21±1.90	7.19±1.90
V2006L	41.12±0.91	140.67±1.35	7.01±0.98
F2108L	40.07±2.13	138.21±0.69	6.78±1.89
S2215Y	39.92±0.12	8.90±0.21	128.1±2.01

Supplementary Table 3. *In vitro* microsomal stability of BC-LI-0186

**Rat liver microsomal stability (%)**	
NADPH (+)	NADPH (-)
10.80 ± 0.02	114.06 ± 0.01

Supplementary Table 4. *In vivo* anti-tumor effect of BC-LI-0186

Group (n=6)	Doses (mg/kg)	Tumor volume$^{(Vt-Vo)\dagger}$						
		Day 0	2	5	7	9	12	14
Vehicle	0	0.0 ± 0.0	29.7 ± 4.0	96.1 ± 2.1	145.4 ± 4.4	216.2 ± 33.6	367.4 ± 36.6	474.7 ± 46.3
BC-LI-0186	20	0.0 ± 0.0	15.8 ± 2.0 *	44.3 ± 5.8 **	92.9 ± 10 **	151.7 ± 16.5 **	221.2 ± 24.5 ***	290.7 ± 43.7 ***
			46.8%‡	53.9%	36.1%	29.8%	39.8%	38.8%
Rapamycin	1	0.0 ± 0.0	27.2 ± 1.6	81.8 ± 1.2 *	118.7 ± 1.8 *	177.5 ± 12.1 *	302.0 ± 21.6 **	385.4 ± 42.5 **
			8.4%	14.9%	18.4%	17.9%	17.8%	18.8%

Significant figures(t-TEST) : * $p<0.05$, ** $p<0.01$, *** $p<0.001$ (vs Vehicle)

† *△t= Vt-Vo, Vt(Measurement of the tumor volume), Vo(Initial tumor volume)*

‡Inhibition Rate(%, vs Vehicle Control)

Supplementary Table 5. Antibody information for western blot

Antibody	Cat. No.	Company	Dilution
Phospho-mTOR (Ser2448)	#2971	Cell signaling technology	1;1,000
mTOR	#2972	Cell signaling technology	1;1,000
phospho-S6Kinase (Thr 389)	#9205	Cell signaling technology	1;1,000
S6Kinase	#9202	Cell signaling technology	1;1,000
Phospho-AKT (Ser 473)	#9271	Cell signaling technology	1;1,000
AKT	#9272	Cell signaling technology	1;1,000
LC3B	#2775	Cell signaling technology	1;1,000
RagB (D18F3)	#8150	Cell signaling technology	1;1,000
Vps34	#4263	Cell signaling technology	1;1,000
mLST8	#3274	Cell signaling technology	1;1,000
Raptor	#2280	Cell signaling technology	1;1,000
FLAG	#8146	Cell signaling technology	1:2,000
Cleaved PARP	#5625	Cell signaling technology	1;1,000
PARP	#9542	Cell signaling technology	1;1,000
Caspase-3	#9662	Cell signaling technology	1;1,000
Cleaved caspase-3	#9661	Cell signaling technology	1;1,000
Phospho-eIF2a (Ser51)	#9721	Cell signaling technology	1;1,000
eIF2a	#9722	Cell signaling technology	1;1,000
Phospho-4EBP1 (Thr37/46)	#9459	Cell signaling technology	1;1,000
4EBP1	#9452	Cell signaling technology	1;1,000
CoxIV	#4850	Cell signaling technology	1;1,000
GM130 (D6B1)	#12480	Cell signaling technology	1;1,000
RagD	A304-301A	Bethyl Laboratories	1:2,000
Myc	A190-105A	Bethyl Laboratories	1:2,000
HA	sc-7392	Santa Cruz biotechnology	1:2,000
Raptor	sc-81537	Santa Cruz biotechnology	1;1,000
ARF1	sc-53168	Santa Cruz biotechnology	1;1,000
RAB1A	11671-1-AP	Proteintech Group	1:2,000
LRS (Leucyl-tRNA synthetase)	NMS-01-0007	Neomics	1:2,000
EPRS (Glutamyl-prolyl tRNA synthetase)	NMS-01-0004	Neomics	1:2,000
LAMP2	ab203224	Abcam	1:2,000
Actin	A5441	Sigma-Aldrich	1:5,000
